# Squirmers with swirl: a model for *Volvox* swimming

**DOI:** 10.1017/jfm.2016.306

**Published:** 2016-05-31

**Authors:** T. J. Pedley, D. R. Brumley, R. E. Goldstein

**Affiliations:** 1 Department of Applied Mathematics and Theoretical Physics, University of Cambridge, Centre for Mathematical Sciences, Wilberforce Road, Cambridge CB3 0WA, UK; 2 Ralph M. Parsons Laboratory, Department of Civil and Environmental Engineering, Massachusetts Institute of Technology, Cambridge, MA 02139, USA; 3 Department of Civil, Environmental and Geomatic Engineering, ETH Zurich, 8093 Zurich, Switzerland

**Keywords:** biological fluid dynamics, micro-organism dynamics, swimming/flying

## Abstract

Colonies of the green alga *Volvox* are spheres that swim through the beating of pairs of flagella on their surface somatic cells. The somatic cells themselves are mounted rigidly in a polymeric extracellular matrix, fixing the orientation of the flagella so that they beat approximately in a meridional plane, with axis of symmetry in the swimming direction, but with a roughly 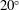 azimuthal offset which results in the eponymous rotation of the colonies about a body-fixed axis. Experiments on colonies of *Volvox carteri* held stationary on a micropipette show that the beating pattern takes the form of a symplectic metachronal wave (Brumley *et al.* *Phys. Rev. Lett.*, vol. 109, 2012, 268102). Here we extend the Lighthill/Blake axisymmetric, Stokes-flow model of a free-swimming spherical squirmer (Lighthill *Commun. Pure Appl. Maths*, vol. 5, 1952, pp. 109–118; Blake *J. Fluid Mech.*, vol. 46, 1971*b*, pp. 199–208) to include azimuthal swirl. The measured kinematics of the metachronal wave for 60 different colonies are used to calculate the coefficients in the eigenfunction expansions and hence predict the mean swimming speeds and rotation rates, proportional to the square of the beating amplitude, as functions of colony radius. As a test of the squirmer model, the results are compared with measurements (Drescher *et al.* *Phys. Rev. Lett.*, vol. 102, 2009, 168101) of the mean swimming speeds and angular velocities of a different set of 220 colonies, also given as functions of colony radius. The predicted variation with radius is qualitatively correct, but the model underestimates both the mean swimming speed and the mean angular velocity unless the amplitude of the flagellar beat is taken to be larger than previously thought. The reasons for this discrepancy are discussed.

## 1 Introduction

**Figure 1.**
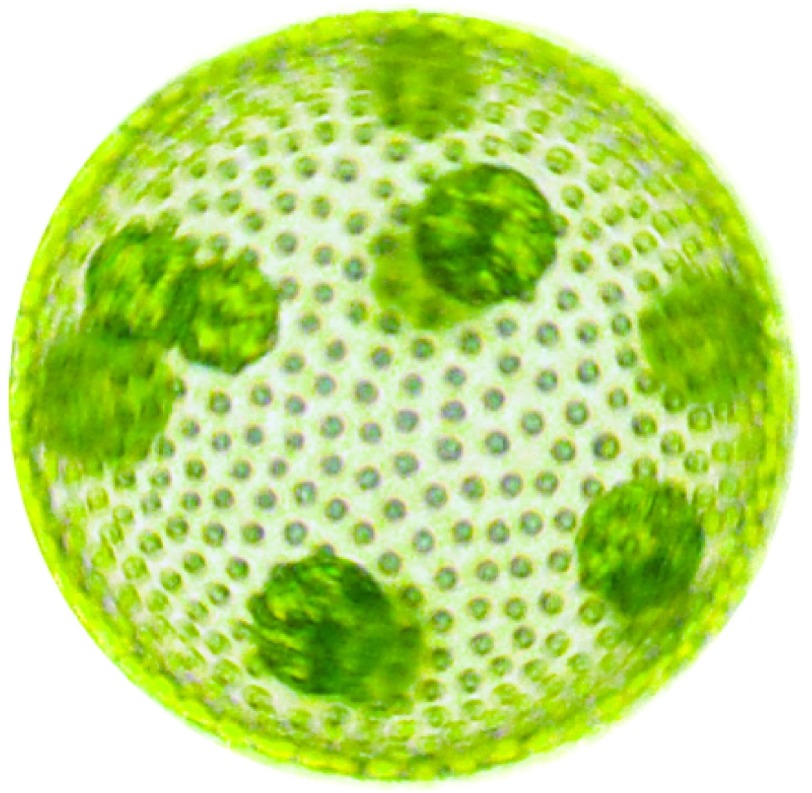
A colony of *Volvox carteri*. Small green dots are the somatic cells on the outside (2000–6000 for *V. carteri*); larger green spheroids are the interior daughter colonies. The photograph is taken from above, as the colony swims upwards towards the camera.

*Volvox* is a genus of algae with spherical, free-swimming colonies consisting of up to 50 000 surface somatic cells embedded in an extracellular matrix and a small number of interior germ cells which develop to become the next generation (figure 1). Discovered by van Leeuwenhoek (1700), who marvelled at their graceful swimming, it was named by Linnaeus (1758) for its characteristic spinning motion. The colony swims in a direction parallel to its anterior–posterior axis thanks to the beating of a pair of flagella on each somatic cell. All flagella exhibit an approximately coplanar, meridional beat, with the power stroke directed towards the rear, i.e. from the north pole towards the south pole, except that the plane of beating is in fact offset from a purely meridional plane by an angle of 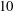
–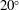. It is believed that this offset causes the observed rotation (Hoops 1993, 1997). The colonies are about 0.3 % denser than water, and swim upwards in still water; this is because the relatively dense interior cells are clustered towards the rear, so when the anterior–posterior axis is deflected from vertical, the colony experiences a restoring gravitational torque that competes with a viscous torque to right the colony on a timescale of 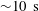. It is remarkable that a typical, free-swimming *Volvox* colony swims in a constant (vertical) direction, suggesting axially symmetric coordination of the flagellar beating, and that it clearly rotates about the axis of symmetry.

### 1.1 Experimental background

**Figure 2.**
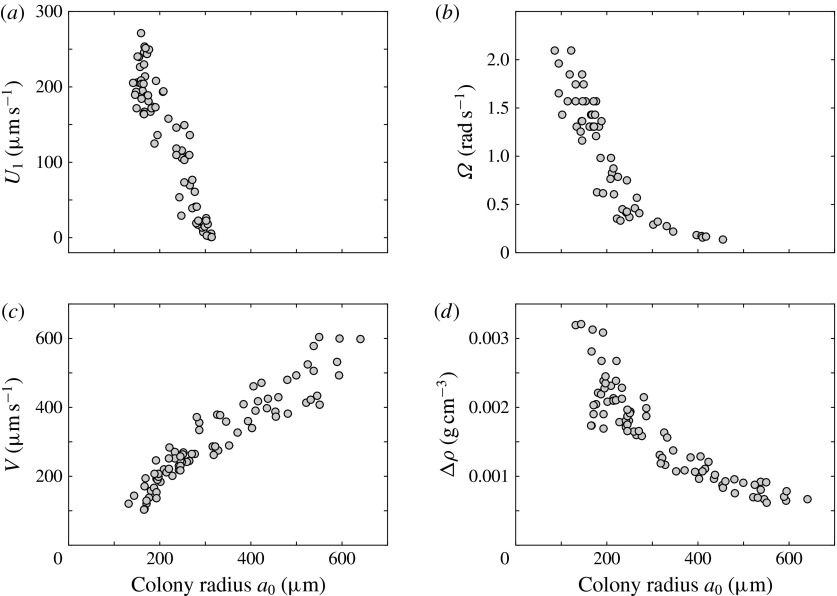
Swimming properties of *V. carteri* as a function of colony radius 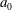. Measured values of the (*a*) upswimming speed 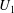, (*b*) angular velocity 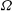 and (*c*) sedimentation speed 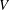, as well as (*d*) the deduced density offset 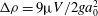 compared with the surrounding medium. Adapted from Drescher *et al.* (2009).

During its 48-h life cycle, the size of a *Volvox* colony increases, though the number and size of somatic cells do not. Thus one would expect the sedimentation speed 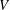 of a colony whose swimming was arrested to increase with colony radius 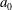, while its upswimming speed 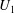 would decrease, both because of the increase in 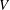 and because, even if it were neutrally buoyant, one would expect the viscous drag to increase with size and hence the swimming speed 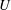 to decrease. Presumably the angular velocity about the axis, 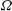, would also decrease. Drescher *et al.* (2009) measured the swimming speeds, sedimentation speeds, and angular velocities of 78, 81 and 61 colonies of *V. carteri*, respectively, ranging in radius from about 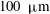 to about 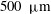. The results are shown in figure 2, where indeed both 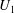 and 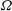 are seen to decrease with 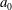, while 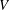 increases. The expected swimming speed if the colony were neutrally buoyant would be 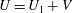 (Solari *et al.* 2006), where linearity is expected because the Reynolds number of even the largest colony is less than 0.1, so the fluid dynamics will be governed by the Stokes equations.

**Figure 3.**
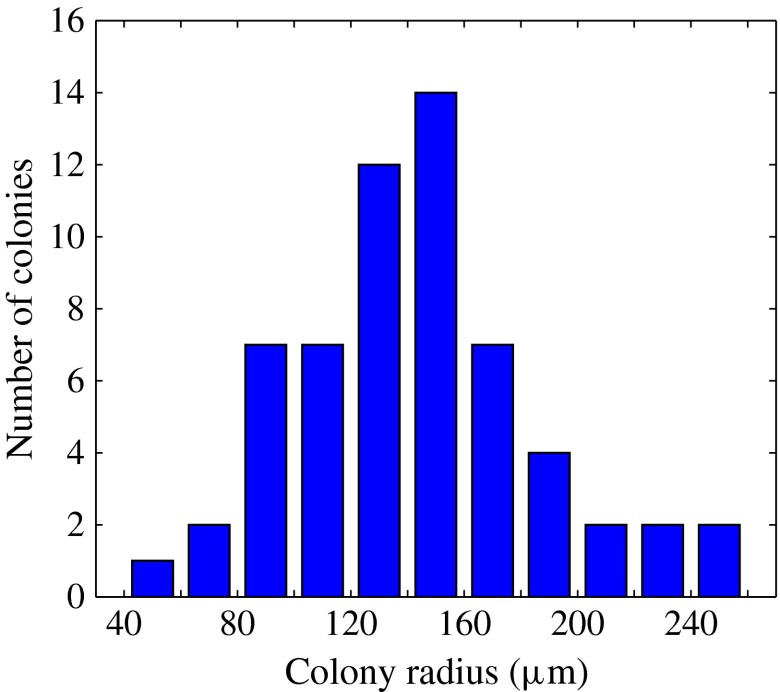
Distribution of colonies by radius, for which the metachronal wave properties are characterised. Adapted from figure 1(*b*) of Brumley *et al.* (2015*a*).

The purpose of this paper is to describe a model for *Volvox* swimming from which both 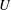 and 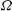 can be predicted, and to compare the predictions with the experiments of figure 2. The input to the model will be the fluid velocities generated by the flagellar beating as measured by Brumley *et al.* (2012, 2015*a*,*b*). Detailed measurements were made of the time-dependent flow fields produced by the beating flagella of numerous *V. carteri* colonies. Individual colonies were held in place on a micro-pipette in a 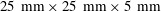 glass observation chamber; the colonies were attached at the equator and arranged so that the symmetry axis of a colony was perpendicular both to the pipette and to the field of view of the observing microscope. The projection of the flow field onto the focal plane of the microscope was visualised by seeding the fluid medium with 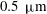 polystyrene microspheres at a volume fraction of 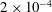, and 30-second-long high-speed movies were taken. The (projected) velocity field was measured using particle image velocimetry (PIV); a total of 60 different colonies were investigated, ranging in radius from 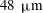 to 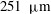 (mean 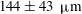), the distribution of which is shown in figure 3.

**Figure 4.**
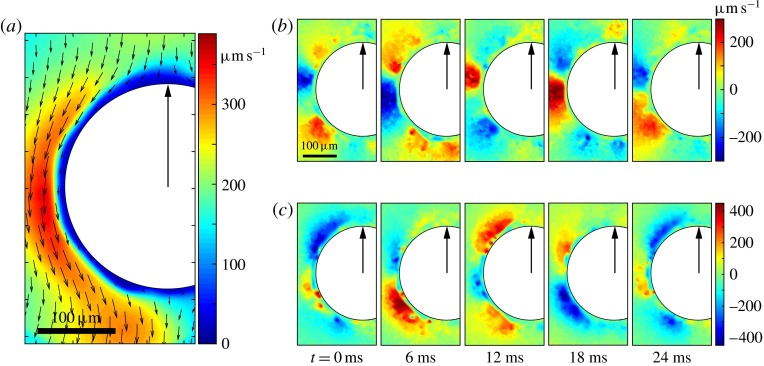
Experimental flow fields. (*a*) Magnitude (colour) and direction (arrows) of the time-averaged velocity field measured with PIV. Radial 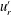 (*b*) and tangential 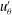 (*c*) components of the unsteady fluid velocity field shown at various times through one flagellar beating cycle. Parts (*a*) and (*b*) are adapted from figures 1(*c*) and (*d*), respectively, of Brumley *et al.* (2015*a*).

One example of the time-averaged magnitude of the velocity distribution is shown in figure 4(*a*). This is a maximum near the equator because the flagellar beating drives a non-zero mean flow past the colony, parallel to the axis of symmetry and directed from front to back. This is consistent with the fact that untethered colonies swim forwards, parallel to the axis.

**Figure 5.**
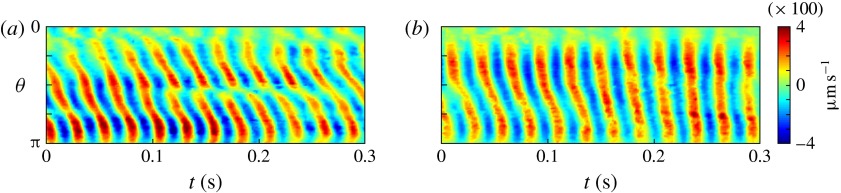
Kymographs of radial 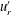 (*a*) and tangential 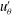 (*b*) velocity around *Volvox* colonies, measured at a radius of 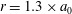.

More interesting are the perturbations to this mean flow. Time-dependent details of the velocity field can be seen in movies S1 and S2 which are available at http://dx.doi.org/10.1017/jfm.2016.306. Close to the colony surface, backwards and forwards motion, driven by the beating flagella, can be clearly seen; further away the flow is nearly steady. Figure 4 contains a series of snapshots showing unsteady components of (*b*) the radial velocity, 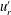, and (*c*) the tangential velocity, 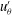. It is immediately evident that the maximum of radial velocity propagates as a wave from front to back, in the same direction as the power stroke of the flagellar beat: a symplectic metachronal wave (Sleigh 1962). This is further demonstrated in figure 5 which shows kymographs of 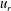 and 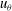 measured at a distance 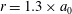 from the colony surface: the propagating wave is clearly seen in figure 5(*a*), which includes evidence of an interesting phase defect, while figure 5(*b*) suggests that the tangential velocity behaves more like a standing wave, dominated by the power stroke near the equator. (The mechanism underlying the coordination of the flagellar beats between the thousands of quite widely spaced somatic cells is itself thought to stem from the fluid mechanical interaction between them. Brumley *et al.* (2015*a*) developed a model for this coordination, as well as for phase defects; it will not be expanded on here.)

Each set of velocity measurements by Brumley *et al.* (2012) are projections onto a single meridional plane. However, the clear axial symmetry of a *Volvox* colony, freely swimming and spinning, indicates that it is reasonable to assume that the flagellar displacement and the consequent velocity fields are also axisymmetric. The fact that the colonies were held fixed means that a force and torque were applied to them while the measurements were being made. This may mean that the flagellar displacements, relative to the colony surface, differed from those for the same colony when swimming freely. The same goes for any constraints felt by a pinned colony due to the proximity of the chamber walls, though this effect is probably small since the largest colonies have diameter around 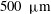, about one tenth of the minimum chamber dimension. We have no direct evidence on these questions, and will assume that the two flagellar beats are the same.

The results of Brumley *et al.* (2012) show that a good fit to the observations of the radial velocity perturbations is given by the following simple form: 1.1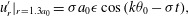 where 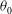 is the polar angle, 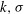 are the wavenumber and frequency of the wave, and 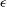 is an amplitude parameter. The mean values of 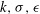 over all of the colonies observed were 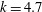, 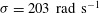, 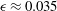. Such data for each colony measured will make up the full input to our model below.

### 1.2 Theoretical background

**Figure 6.**
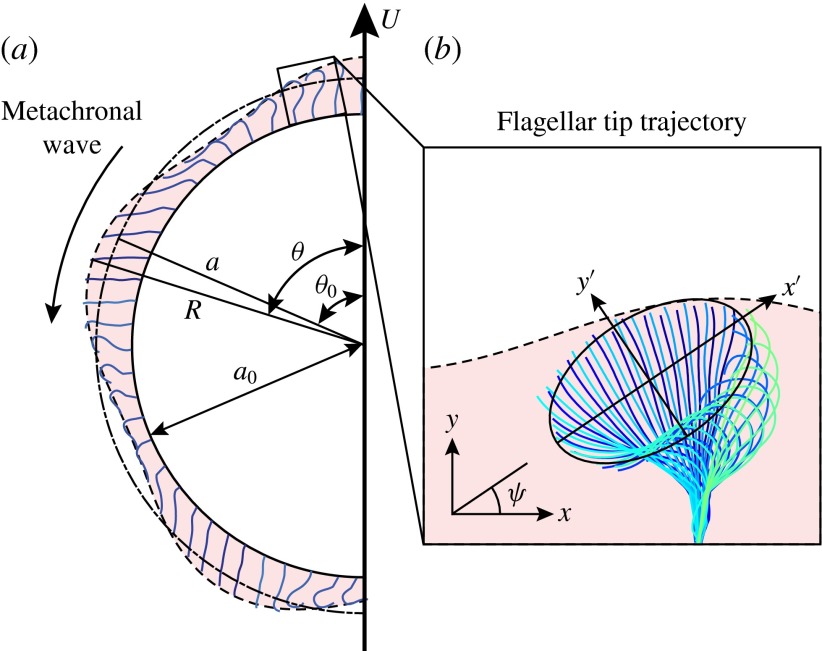
(*a*) Schematic diagram of a spherical *Volvox* colony at one instant in time, with beating flagella and the envelope of flagellar tips. The radius of the extracellular matrix in which the flagella are embedded is 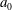. The mean radius of the envelope is 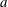; 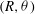 are the coordinates of a surface element whose average position is 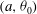. (Adapted from Blake (1971*b*), but replotted with the experimentally determined metachronal wavenumber.) (*b*) Measured tip trajectory over multiple beats of a single *Volvox* flagellum. The trajectory is fitted with an ellipse, which is rotated at an angle 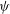 with respect to the local colony surface.

The model will be an extension to the swirling case of the spherical envelope (or ‘squirmer’) model for the propulsion of ciliated protozoa introduced by Lighthill (1952) and Blake (1971*b*). When the surface of a cell is densely covered with beating cilia, as for the protist *Opalina* for example, it is a very good approximation to treat the flow around it as being driven by the displacement of a stretching flexible sheet, attached to the tips of all of the cilia and moving with them. The sheet will undergo radial and tangential wave-like displacements, and it needs to stretch to accommodate temporal variations between the displacements of neighbouring cilia tips (figure 6*a*). In the case of *Volvox carteri* the tips of the beating flagella are not very close together; for a colony of radius 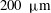, the average spacing between somatic cells is 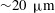, comparable with the flagellar length, 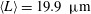 (Brumley *et al.* 2014), so the envelope model may well be somewhat inaccurate. As indicated above, the new feature of our model is the introduction of azimuthal swirl to the envelope model.

The theory will be given in the next two sections, first extending the Lighthill–Blake model to include swirl, and second applying the model to *Volvox* on the basis of the data of Brumley *et al.* (2012). The objective is to calculate the mean swimming speed 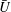 and mean angular velocity 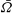, and test the model by comparison with the measurements of Drescher *et al.* (2009). The final section will include a discussion of discrepancies and the model’s limitations.

## 2 Theory for squirmers with swirl

In the original, zero-Reynolds-number, spherical-envelope model of ciliated micro-organisms (Lighthill 1952; Blake 1971*b*), the radial and tangential Eulerian velocity components 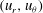 are written as infinite series of eigensolutions of the Stokes equation:

2.1*a*
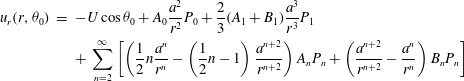

2.1*b*
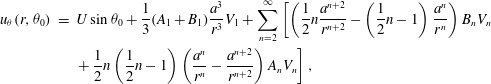

assuming axial symmetry. Here 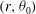 are spherical polar coordinates, the 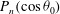 are Legendre polynomials, and 2.2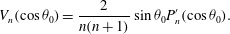 A trace of a typical flagellar beat is shown in figure 6(*b*), adapted from Brumley *et al.* (2014), where it can be seen that the trajectory of the tip is approximately elliptical, with centre about two-thirds of the flagellar length from the surface of the extracellular medium. Thus, 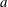 is taken to be the mean radius of a flagellar tip, so we take 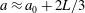, where 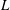 is the length of a flagellum. With the origin fixed at the centre of the sphere, 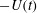 is the speed of the flow at infinity (i.e. 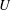 is the instantaneous swimming speed of the sphere). If the sphere is taken to be neutrally buoyant, it experiences no external force, so the Stokeslet term must be zero, and 2.3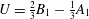 (Blake 1971*b*). Corresponding to the velocity field (2.1), the velocity components on the sphere 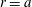 are 2.4*a*,*b*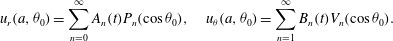 From this we can see that 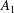 should be zero, because it corresponds to longitudinal translation of the centre, which is incorporated into 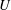. However, we follow Lighthill (1952) and not Blake (1971*b*) in retaining a non-zero 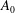. Blake wished to prohibit any volume change in his squirmers, which is of course physically correct, although if there really were an impenetrable membrane covering the flagellar tips and if, say, all of the flagella beat synchronously, the envelope of their tips would experience a small variation in volume, so 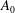 should not be zero. Our choice of sinusoidal velocity and displacement wave, equations (1.1) and (3.1) below, in fact requires a non-zero 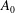. It turns out that for the parameter values applicable to *Volvox* the presence or absence of this term makes little difference to the predictions of mean swimming speed, and it does not affect the angular velocity anyway.

The surface velocities in (2.4) must in fact be generated by the motion of material elements of the spherical envelope, representing the tips of the beating flagella. In the Lighthill–Blake analysis, the envelope is represented by the following expressions for the Lagrangian coordinates 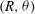 of the material elements:

2.5*a*
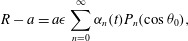

2.5*b*
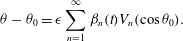

The functions 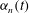 and 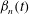 are supposed to be oscillatory functions of time with zero mean, and the amplitude of the oscillations, 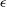, is taken to be small. The most intricate part of the theory is the calculation of the 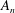 and 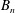 in (2.4) in terms of the 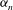 and 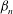 in (2.5). This will be outlined below.

The new feature that we introduce in this paper is to add axisymmetric swirl velocities and azimuthal (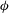) displacements to the above. The 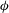 component of the Stokes equation is 2.6
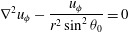
 and the general axisymmetric solution that tends to zero at infinity is
2.7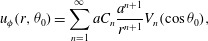 equal to 2.8
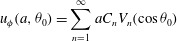
 on 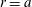. Now the total torque about the axis of symmetry is 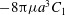 and, since the sphere is our model for a free-swimming *Volvox* colony, this, like the total force, must be zero, i.e. 2.9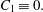 Analogous to (2.5), the 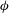 displacement of the material point 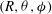 on the spherical envelope is taken to be 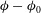 where 2.10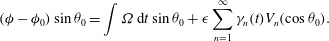 Here 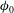 is fixed on the rotating sphere, and 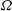 is the instantaneous angular velocity of the sphere. The general solution for a squirmer with non-axisymmetric (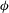-dependent) squirming and swirling has been given in terms of vector spherical harmonics by Ghose & Adhikari (2014), Pak & Lauga (2014), Felderhof (2016) and Felderhof & Jones (2016). They all calculated the body’s translational and angular velocities corresponding to an arbitrary distribution of velocities on 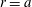, but only Felderhof related the surface velocities to Lagrangian displacements of surface elements.

The relations between the Eulerian velocities (2.1), (2.7) and the Lagrangian displacements (2.5), (2.10), from which 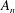, 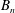, 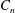 and 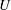, 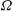 are to be derived from 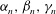, are 2.11*a*-*c*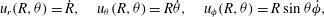 where an overdot represents the time derivative. Blake (1971*b*) performed the analysis for the 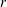 and 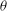 velocities; here we illustrate the method by deriving the relation between the 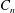 and the 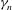.

The analysis is developed in powers of the amplitude 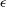, so we take

2.12*a*
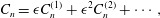

2.12*b*
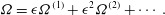

At leading order, 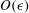, equations (2.11c) and (2.10) give 2.13*a*,*b*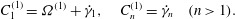 Immediately, therefore, we see from (2.9) that 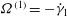, which has zero mean, so the mean angular velocity, like the mean translational speed, is 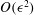. At second order, the fact that 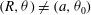 is important in the expression for the velocity field: 2.14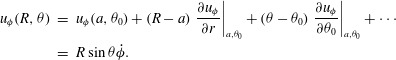 Substituting for 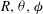 gives 2.15
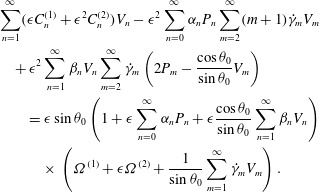
 Taking the 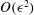 terms in this equation, multiplying by 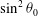 and integrating from 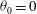 to 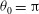 (recalling that 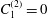), gives the following explicit expression for 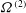: 2.16
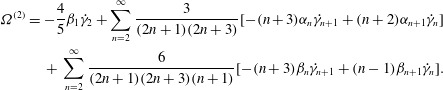
 (Some of the required integrals of products of 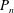 and 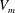 are given in appendix A.) The corresponding result for the second-order term in the translational velocity is
2.17
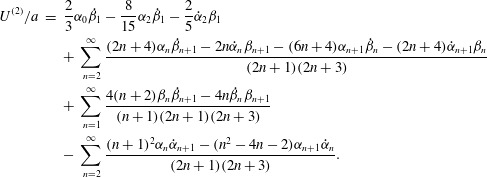
 This is the formula given by Blake (1971*b*), except that he omitted the term involving 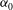 which Lighthill (1952) included; Lighthill omitted some of the other terms.

A shortcut to predicting 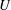 and 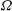 was proposed by Stone & Samuel (1996), following Anderson & Prieve (1991). They used the reciprocal theorem for Stokes flow to relate the translation and rotation speeds of a deformable body with non-zero surface velocity 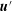 to the drag and torque on a rigid body of instantaneously identical shape, and derived the following results for a sphere of radius 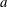, surface 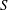:

2.18*a*
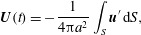

2.18*b*
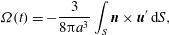

where 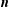 is the outward normal to the sphere. From the first of these (2.3) follows. It turns out not to be so simple to use these results for squirmers with non-zero radial deformations, because of the need to calculate the drag to 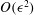 for the rigid deformed sphere.

## 3 Application to *Volvox*

In order to apply the above theory to *Volvox*, we need to specify the 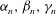. This will be done by making use of the experimental results on the metachronal wave by Brumley *et al.* (2012), which led to (1.1) for the radial velocity distribution on the envelope of flagellar tips, plus assumptions about the tangential and azimuthal displacements. Following (1.1), we write the radial displacement as 3.1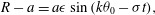 where 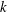 is the wavenumber, 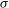 the radian frequency, and 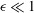. Observations of flagellar beating show that a flagellar tip moves in an approximately elliptical orbit (see figure 6*b*). Thus, we may write 3.2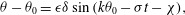 where figure 6(*b*) suggests 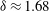 and the phase difference 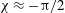. The observation that the plane of beating of the flagella is offset by 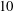
–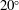 from the meridional plane suggests that the functional form of the 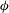 displacement, relative to the rotating sphere, is also given by (3.2), multiplied by a constant, 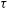, equal to the tangent of the offset angle. Together, then, equations (2.5), (2.10), (3.1) and (3.2) give

3.3*a*
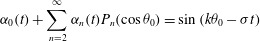

3.3*b*
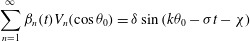

3.3*c*
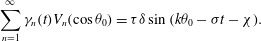

It can be seen immediately that 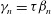, so only (3.3*a*) and (3.3*b*) need to be solved for 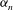 and 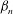. To do this requires expressions for 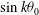 and 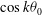 as series of both 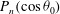 and 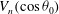:

3.4*a*
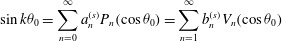

3.4*b*
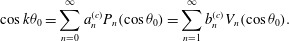

The results for 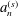 etc. (see appendix B) are

3.5*a*
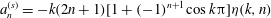

3.5*b*
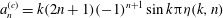

3.5*c*
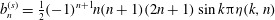

3.5*d*
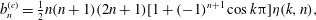

where 3.6
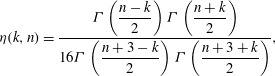
 and 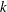 is assumed not to be an integer. It then follows from (3.3) that

3.7*a*
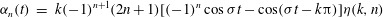

3.7*b*
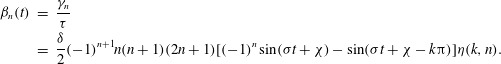

Now we can put (3.7) into (2.16) and (2.17), take the mean values and obtain final results for the second-order contributions to the mean angular and translational velocities: 3.8
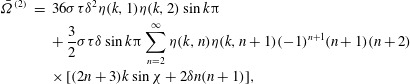

3.9
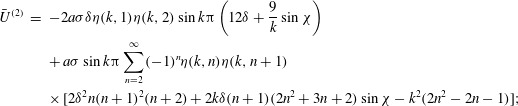
 note that non-zero 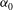 makes no difference to 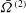. We may also note that calculations are made easier by recognising that
3.10
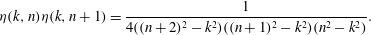


We now put in parameter values obtained from the experiments of Brumley *et al.* (2012) and compare the predicted values of 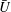 and 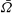 with the measurements of Drescher *et al.* (2009). Rather than merely using the average values of 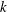 and 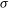 quoted by Brumley *et al.* (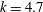, 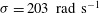), we use the individual values for each of the 60 *Volvox* colonies from which the averages were obtained, together with their radii 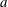. We also need the value of the dimensionless amplitude 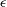. As discussed above, the recorded radius 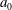 is the radius of the surface of the extracellular matrix in which the somatic cells are embedded, and 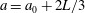 and, hence, 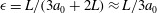 (noting the typical orbit in figure 6*b*). Solari *et al.* (2011) have shown that flagellar length, as well as colony radius, increases as a colony of *V. carteri* or *V. barberi* ages. The values of 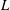 (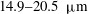) and 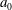 quoted by them give values of 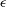 between 0.029 and 0.038; thus, we may be justified in choosing 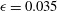 as normal. We also use the value of 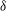 (1.68) quoted above, although trajectories of flagellar tips measured by Brumley *et al.* (2014) show a range of values of 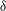 from 1.45 to 1.86. Moreover, we use 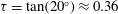 although we do not have measurements of the offset angle for individual colonies.

**Figure 7.**
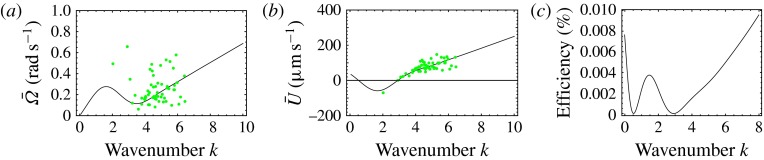
Predicted values of (*a*) mean angular velocity 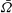, (*b*) mean swimming speed 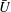 and (*c*) mechanical efficiency, 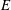, as functions of the metachronal wavenumber 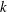. Green dots are predictions of the squirmer model using the individually measured parameters for each of the 60 *Volvox* colonies. The solid lines are the predictions using the mean properties (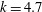, 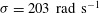). Other parameters include 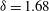, 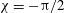, 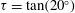. Here the mean amplitude is 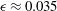, equivalent to flagella length 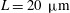.

The results for 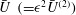 and 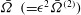 are plotted against 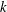 in figure 7, where the dots use the individual values of 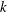, 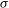 and 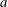 in each of the 60 *Volvox* colonies measured by Brumley *et al.* (2015*a*). The continuous curve uses the mean values of 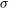 and 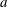; all results assume a flagellum of length 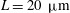, and a mean value of 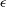 of 0.035. It is interesting that 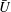 and, to a lesser extent, 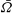 increase regularly with 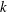 over the range of measured values, but would vary considerably for lower values, even resulting in negative mean swimming speeds.

Also plotted, in figure 7(*c*), is the mechanical efficiency 3.11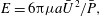 where 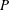 is the instantaneous rate of working of the stresses at the surface of the sphere, 3.12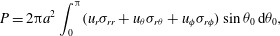 and 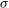 is the stress tensor. The formula for 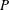 in the absence of swirl was given by Blake (1971*b*, (9)), the additional, third, term due to swirl is equal to 3.13
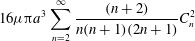
 (see also Pak & Lauga 2014). Figure 7(*c*) shows a local maximum of 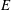 at 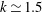, corresponding to negative swimming speed, which may therefore be discounted. For 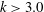, however, the efficiency increases with 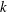. According to this model, then, it appears that the swimming mode of *Volvox* did not come about evolutionarily through energetic optimisation.

**Figure 8.**
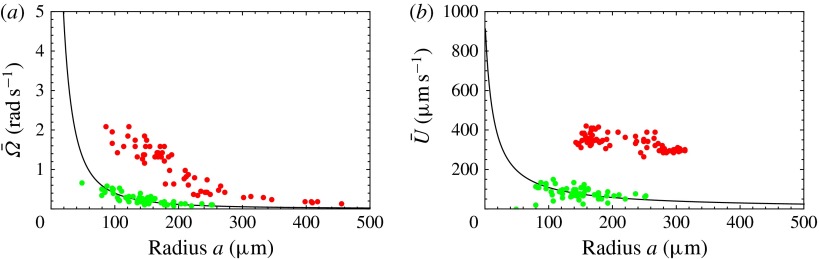
Predicted and measured values of (*a*) mean angular velocity 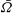 and (*b*) mean swimming speed 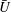, as functions of colony radius. Green dots are predictions of this model, red dots are measurements (on a different population of colonies) by Drescher *et al.* (2009) (cf. figure 2). The solid line is the prediction from mean properties of the 60 colonies whose metachronal wave data have been used.

We plot the calculated 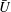 and 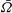 against 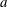 in figure 8. The green points represent colony-specific predictions using data from Brumley *et al.* (2015*a*) and the continuous curves correspond to the mean values of 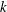, 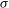 and 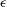 referred to above. The red points represent the experimental values measured by Drescher *et al.* (2009), again using the individual values of 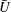, 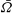 and 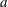 for each of the colonies measured (data kindly supplied by Dr Knut Drescher) rather than an average value. As noted in the introduction, with reference to figure 2, because the above theory assumes neutral buoyancy, the value quoted for 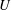 is the sum of the actual upwards swimming speed 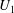 and the sedimentation speed 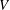 of an inactive colony of the same radius.

**Figure 9.**
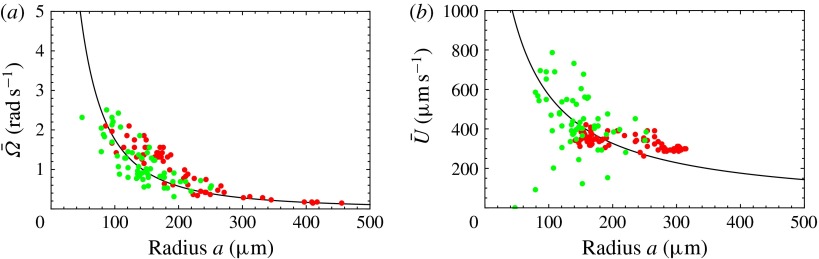
Same as figure 8 but with mean 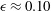 (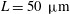).

**Figure 10.**
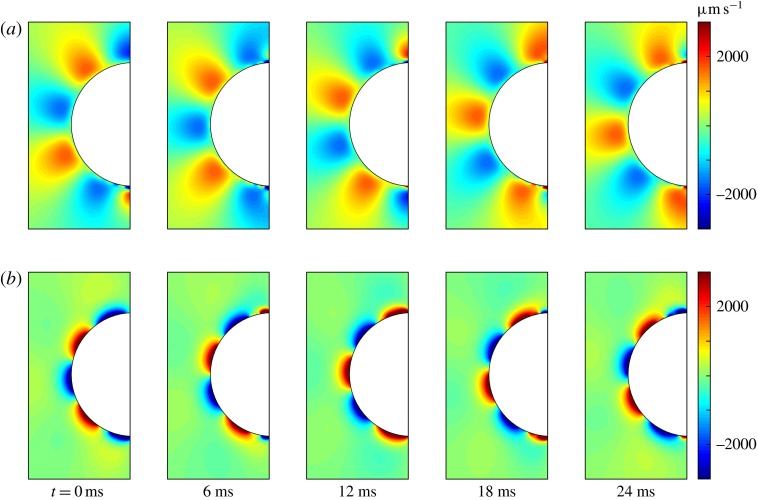
Squirming flow fields. Radial (*a*) and tangential (*b*) components of the fluid velocity field shown at various times through one flagellar beating cycle. The metachronal wave properties ((3.1) and (3.2)) are the same as for the average *Volvox* colony (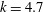, 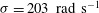, 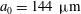) and other parameters correspond to measured flagella and their trajectories (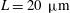, 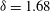, 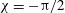).

In figure 8, the predictions for both 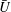 and 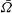 are significantly below the measured values, though the trend with increasing radius is similar. If we had taken the flagellar length 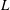 to be 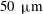 instead of 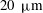, the agreement would seem to be almost perfect (figure 9). In the next section we discuss in more detail aspects of the model that may need to be improved.

**Figure 11.**
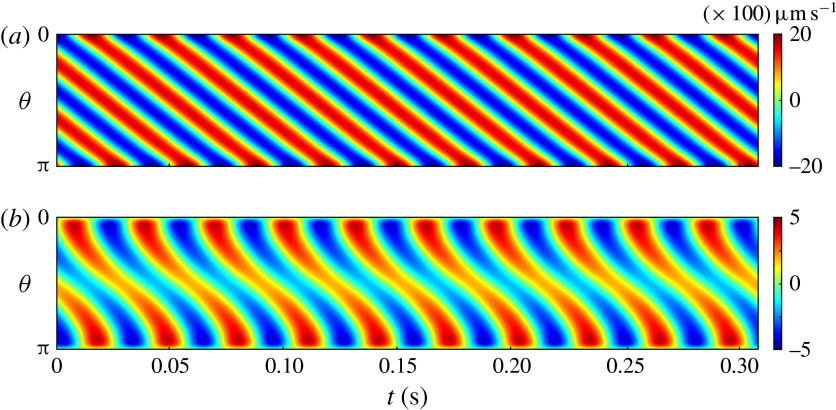
Squirmer kymographs. Radial 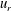 (*a*) and tangential 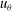 (*b*) components of the flow, as functions of polar angle 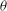 and time 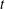, computed at the fixed radius (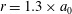). Other parameters are the same as in figure 10.

In addition to calculating 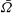 and 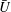 we can use the squirmer model to compute the time-dependent velocity field, for comparison with the measurements in figures 4 and 5. Figure 10 shows the radial and tangential velocities as functions of position at different times during a cycle, for the mean values of 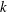 (4.7), 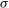 (203 rad s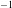) and 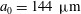. Both velocity components show the metachronal wave, which is not surprising since that was used as input from (3.1) and (3.2). The figure also indicates that the tangential velocity component decays more rapidly with radial distance than the radial component. Calculated kymographs of 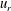 and 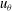 at 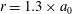 are shown in figure 11, and can be compared with figure 5. There is good qualitative agreement between figures 10 and 11 and figures 4 and 5. Unlike the mean velocity, however, which is lower than measured, the amplitude of the calculated 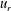 or 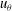 oscillations, scaling as 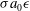 from (2.11*a*-*c*) and (3.1), is about 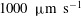, significantly larger than the measured value of about 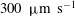 (figure 5).

## 4 Discussion

The main discrepancy between the theoretical predictions of this paper and the experimental observations of Drescher *et al.* (2009) is that, although the maximum fluid velocity during a cycle, for the experimental parameter values, is much larger in the model than measured, the predicted mean velocity and angular velocity are significantly smaller than measured.

The envelope model is clearly a great oversimplification, because even in the context of single-celled ciliates, the cilia tips do not form a continuous surface at all times. Not only may there be wide spaces between neighbouring tips, but also some tips may, during their recovery stroke, be overshadowed by others in their power stroke, so the envelope is not single-valued (Brennen & Winet 1977). The latter is not a problem for *V. carteri*, because the flagellar pairs are more widely spaced, but that in itself adds to the former difficulty. Blake (1971*b*) argued that the envelope model would be a better approximation for symplectic metachronal waves than for antiplectic ones, because the tips are closer together during the power stroke, when their effect on the neighbouring fluid is greatest; this is especially true for a ciliate such as *Opalina*, but is less compelling in the case of *V. carteri*, for which typical cell (and, hence, flagellar) spacings are roughly equal to the flagellar length. The wide spacing between flagellar tips means that much of the ‘envelope’ is not actively engaged in driving fluid past the surface, and fluid can leak back between neighbours, so one would expect the model to overestimate the fluid velocity, as it does if one considers the maximum instantaneous radial or tangential velocity. As reviewed elsewhere (Goldstein 2015), the volvocine algae include a range of species with differing interflagellar distances, some of which are significantly smaller than in *V. carteri*, and one can anticipate that future studies of those species may shed further light on the validity of the envelope model.

**Figure 12.**
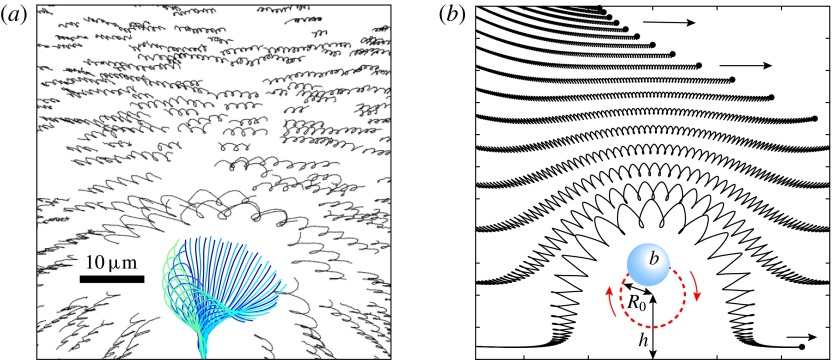
Particle paths in the vicinity of a flagellum. (*a*) Trajectories of 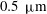 passive tracers near an isolated *Volvox* flagellum held with a glass micropipette. The tracked flagellar waveform from several beats is also shown. (*b*) A sphere of radius 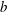 moving in a circular trajectory above and perpendicular to a no-slip boundary produces a time-dependent flow, which closely mimics that of a real flagellum. This simulation of 100 beats shows particle paths from various initial positions, and corresponds to 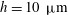, 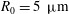, 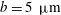.

Why, therefore, is the mean velocity underestimated? It seems likely that the difference lies in the fact that each flagellum beats close to the no-slip surface of the extracellular matrix in which the somatic cells are embedded. In the power stroke, a flagellum is extended and its outer parts, in particular the tip, set neighbouring fluid particles in motion, over a range of several flagellar radii, at about the same speed as the tip. During the recovery stroke, on the other hand, the flagellum is much more curved, and the outer part remains roughly parallel to the colony surface (Blake 1972). Thus the drag exerted by the outer part of the flagellum on the fluid will be reduced by a factor approaching two compared with the power stroke. Moreover, this outer part is relatively close to the colony surface, and the no-slip condition on that surface will prevent fluid particles from moving at the same speed as the tip except very close to it. Both these factors mean that, although every element of the beating flagellum oscillates with zero mean displacement, the fluid velocities that it generates do not have zero mean.

As part of the experiments reported by Brumley *et al.* (2014), movies were taken of the motion of microspheres in the flow driven by a single beating flagellum on an isolated *V. carteri* somatic cell fixed on a micropipette. Experimental details are given briefly in appendix C. One of these movies is reproduced in movie S3, in which the difference between the fluid particle displacements in power and recovery strokes can be clearly seen. The trajectories of a number of the microspheres are shown in figure 12(*a*). Movie S4 and figure 12(*b*) show particle trajectories calculated from a very simple model (see appendix C), which consists of a small spherical bead following a circular orbit perpendicular to a nearby rigid plane (such an orbiting bead model of a beating flagellum has been used extensively in recent years (Lenz & Ryskin 2006; Vilfan & Jülicher 2006; Niedermayer *et al.* 2008; Uchida & Golestanian 2011; Brumley *et al.* 2012, 2015*a*; Bruot & Cicuta 2016)). The similarity between the measured and computed trajectories is clear.

It is therefore evident that the net tangential velocity excess of the power stroke over the recovery stroke of *Volvox* flagella will be 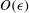, so the mean velocity generated will be 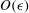 not 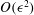 as obtained from our squirmer model. That may be a more important limitation of the model than the wide spacing of the flagella. What is required, in future, is a detailed fluid dynamic analysis of an array of beating flagella on the surface of a sphere. This will be an extension of the so-called sublayer model of Blake (1972) and Brennen & Winet (1977), in which each cilium is represented as a linear distribution of Stokeslets whose strengths can be estimated using resistive force theory, or calculated more accurately as the solution of an integral equation using slender-body theory, taking account of the no-slip boundary by including the Stokeslet image system as derived for a planar boundary by Blake (1971*a*). This model is currently being developed.

Three other assumptions in the theory of this paper should be discussed. First is the choice of a sine wave to represent the displacement of the flagella tips ((3.1) and (3.2)). The choice necessitates some intricate calculations (§ 3 and appendix B) and it could be argued that the measurements of Brumley *et al.* (2012) are not sufficiently refined to justify it. Blake (1971*b*), among others, proposed that four terms in the Legendre polynomial expansions (2.4) would be accurate enough. Moreover, that would avoid the problem of non-zero values for 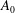 and 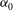. However, a sine wave still seems the most natural choice for a propagating wave, and we have assumed it accordingly.

Another choice made here is to truncate the expansions of derived quantities at 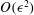, which is likely to lead to errors at larger values of 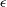 (Drummond 1966); however, even for figure 9, the assumed value of 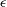 was less than 0.1, so this is unlikely to cause a significant error in figure 8. A third assumption in this paper is that the elliptical trajectory of each flagellar tip has its major axis parallel to the locally planar no-slip colony surface. In fact, it will in general be at a non-zero angle 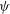 to that surface (figure 6*b*). In that case the calculation becomes somewhat more cumbersome but no more difficult, as outlined in appendix D. If we choose 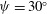, for example, the results for 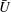 and 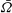 are negligibly different from those in figure 8. The assumption that 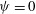 is therefore not responsible for the discrepancy between theory and experiment in that figure.

## Supplementary movies

For supplementary material accompanying this paper visit http://dx.doi.org/10.1017/S0022112016003062. click here to view supplementary material

## Appendix A. Integrals required in the derivation of (2.16)

We seek to evaluate

A 1

and

A 2

where  is defined by (2.2), using the standard recurrence relations and differential equation for Legendre polynomials:

A 3

A 4

A 5

 Here a prime means  and we do not explicitly give the -dependence of . From (A 1),

A 6

where

A 7

Hence,

A 8

From (A 2),

A 9

## Appendix B. Proof of (3.5*a*)

We prove by induction the first of the formulae in (3.5); proofs of the others are similar. Let

B 1

so that

B 2

from the first of (3.4*a*). The result we seek to prove is

B 3

where  is given by (3.6). From (B 1) and (A 4), we have

B 4

Now suppose that (B 3) is true for  and , for all , substitute it into the right-hand side of (B 4), and after some algebra indeed obtain (B 3) with  replaced by . The induction can be shown to start, with  and , using the standard identities

B 5

B 6

 Thus, (B 3) and hence (3.5*a*) are proved.

## Appendix C. Flagellar flow fields

To investigate the time-dependent flow fields produced by individual eukaryotic flagella, Brumley *et al.* (2014) isolated individual cells from colonies of *V. carteri*, captured and oriented them using glass micropipettes, and imaged the motion of  polystyrene microspheres within the fluid at 1000 f.p.s. One such movie is included as movie S3, which shows the time-dependent motion of these passive tracers in the vicinity of the beating flagellum. Using custom-made tracking routines, we identify the trajectories of the microspheres, and these are shown in figure 12(*a*), together with the tracked flagellar waveform over several beats. Tracer particles in the immediate vicinity of the flagellar tip exhibit very little back flow during the recovery stroke.

We consider now the flow field produced by a simple model flagellum, which consists of a sphere of radius  driven at a constant angular speed  around a circular trajectory of radius , perpendicular to an infinite no-slip boundary. The trajectory of the sphere is given by

C 1

where . The velocity of the particle is then

C 2

The force that this particle imparts on the fluid is given by

C 3

We know that , and therefore the time-dependent force exerted on the fluid is

C 4

The fluid velocity  at position  is expressed in terms of the Green’s function in the presence of the no-slip boundary condition (Blake 1971*a*):

C 5

where

C 6

and

C 7

C 8

C 9

 For a passive tracer with initial position  at , its trajectory can be calculated according to

C 10

Numerical solutions of (C 10) are shown in figure 12(*b*) for various initial positions. The parameters used are designed to mimic those of real *Volvox* flagella (, ). A sphere of radius  is used, though we emphasise that strictly speaking this does not come into contact with the plane. The finite value of  is used simply to generate variable drag as a function of height, in order to produce a net flow. In addition, the particle trajectories are independent of the speed of the sphere, and so the results in figure 12(*b*) would be unchanged if the sphere were instead driven by either a constant force, or by a phase-dependent term.

## Appendix D. Rotated ellipse

In this section, we consider the case in which the elliptical trajectory of the flagellar tip is rotated at an angle  with respect to the surface of the *Volvox* colony. In this case, (3.1) and (3.2) can be generalised to become

D 1

D 2

 The series expansions for these are then given by

D 3

D 4

 and  as before. Equations (D 3) and (D 4) need to be solved for  and , but this follows easily by linearity using the solutions in (3.7*a*) and (3.7*b*), together with appropriate transformations in . Calculation of  and  is more challenging, but after considerable algebra, we find the following:

D 5

and

D 6

Note that (D 5) and (D 6) reduce to (3.8) and (3.9) respectively when .
