# Supplementary material for: Squirmers with swirl: a model for Volvox swimming
Source: J Fluid Mech. 2016 May 31;798:165–86. doi: 10.1017/jfm.2016.306 (PMC5070036; doi:10.1017/jfm.2016.306)
Supplement: Supplementary file 1 [file S0022112016003062sup.zip › FLM1600306_Movies/flm_1600306_Captions.rtf]

Movie 1
Radial component of fluid velocity field, measured with PIV, as a function of polar angle   and time (cf.  Figure 4b).
Movie 2
Tangential component of fluid velocity field, measured with PIV, as a function of polar angle   and time (cf.  Figure 4c).
Movie 3
Observed particle motions in the vicinity of a single beating flagellum on an isolated Volvox somatic cell (cf.  Figure 12a).
Movie 4
Computed particle trajectories generated by a microsphere moving in a circular orbit above and perpendicular to a no-slip boundary (cf.  Figure 12b).
